# Multinucleate cell angiohistiocytoma: an uncommon cutaneous tumor^☆^^☆☆^

**DOI:** 10.1016/j.abd.2019.10.005

**Published:** 2020-05-11

**Authors:** Anderson Alves Costa, Glaucia Ferreira Wedy, Walter Belda Junior, Paulo Ricardo Criado

**Affiliations:** a Department of Dermatology, Universidade de São Paulo, São Paulo, SP, Brazil; b Department of Dermatology, Faculdade de Medicina do ABC, Santo André, SP, Brazil

**Keywords:** Case reports, Giant cells, Histiocytoma, Lower extremity, Neoplasms, Vascular tissue

## Abstract

Multinucleate cell angiohistiocytoma is a rare, benign vascular proliferation of unknown etiology. It occurs mainly in middle-aged women and usually affects the acral regions; the lesions appear as discrete, grouped, and asymptomatic violaceous papules. Histopathology shows proliferation and dilated small vessels in the papillary dermis, fibrous stroma with thickened collagen bundles, and multinucleated giant cells. To date, there are approximately 140 cases described in the indexed literature. This report presents the case of a 62-year-old woman with a typical clinical condition, who chose not undergo treatment, considering the benign character of her illness. The clinical and immunohistological aspects of this unusual dermatological entity are emphasized.

## Introduction

Multinucleate cell angiohistiocytoma (MCAH) is a rare, benign vascular proliferation of the skin, whose cause was unknown until recently. Currently, approximately 140 cases have been described in indexed literature (PubMed/MEDLINE). This report presents another clinical case of MCAH studied under immunohistological basis and presents a review of the relevant literature that delineates the prominent clinicopathological features of this unusual entity, which is often confused with dermatofibroma in the clinical setting.

## Case report

**Figure 1:**
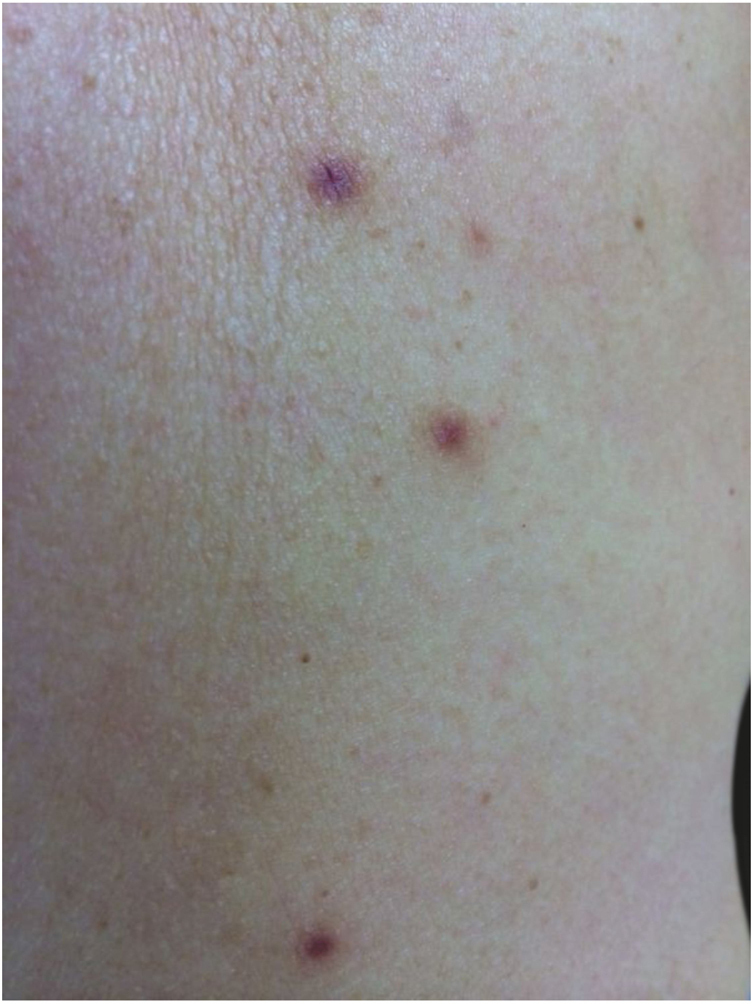
Cutaneous erythematoviolaceous flatted papules ranging from 3 to 10 mm in diameter, arranged on the dorsum.

A 62-year-old Caucasian woman was referred to this clinic by her general physician due the onset of cutaneous lesions on left arm and right forearm eight months ago. The patient's past medical history was unremarkable. An extensive panel of routine blood exams revealed no significant abnormalities, including negative anti-HIV serology. She denied previous solid organ transplant or use of immunosuppressive drugs. The lesions were asymptomatic. In the last two months, she noticed a new group of lesions on her abdomen, right thigh, and back. On dermatological examination, asymmetric painless erythematoviolaceous papules were observed, with 3–10 mm in diameter (Fig. 1).

**Figure 2:**
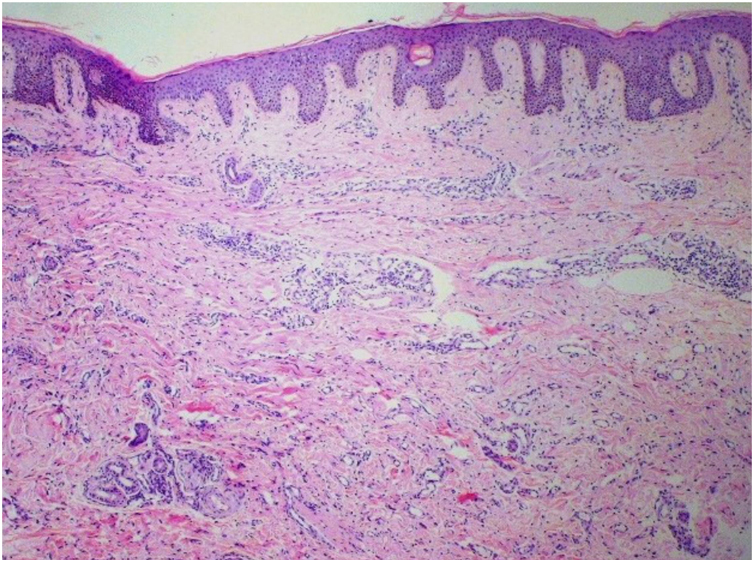
Regular acanthosis in the epidermis and overall increased cellularity were present throughout the dermis, as well as an increased number of dilated blood vessels in the upper and mid dermis, lymphohistiocytic infiltrate, and thicker collagen bundles (Hematoxylin & eosin, ×40).

**Figure 3:**
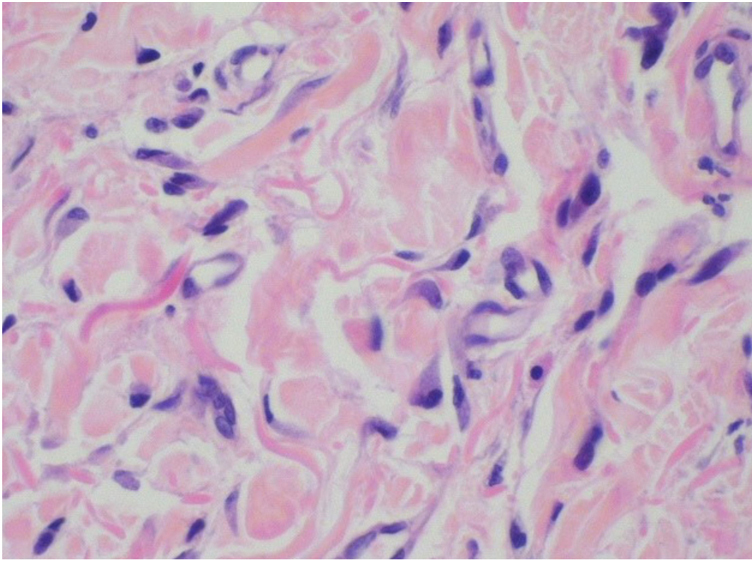
Characteristic irregular multinucleate cells of MCAH, typified by angular borders and multiple potentially hyperchromatic nuclei in the dermis and enlargement of endothelial nuclei in the capillaries (Hematoxylin & eosin, ×400).

**Figure 4:**
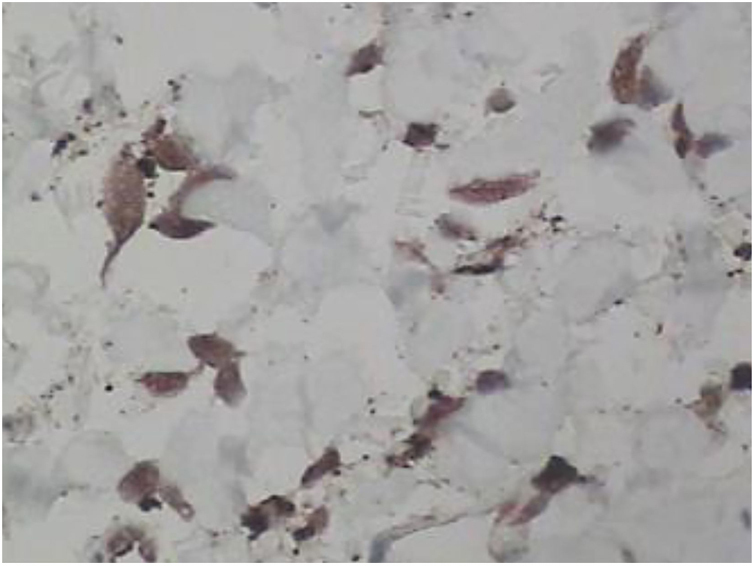
Immunohistochemistry study. Histiocytic and multinucleated cells are positive for Factor XIIIa (×400).

**Figure 5:**
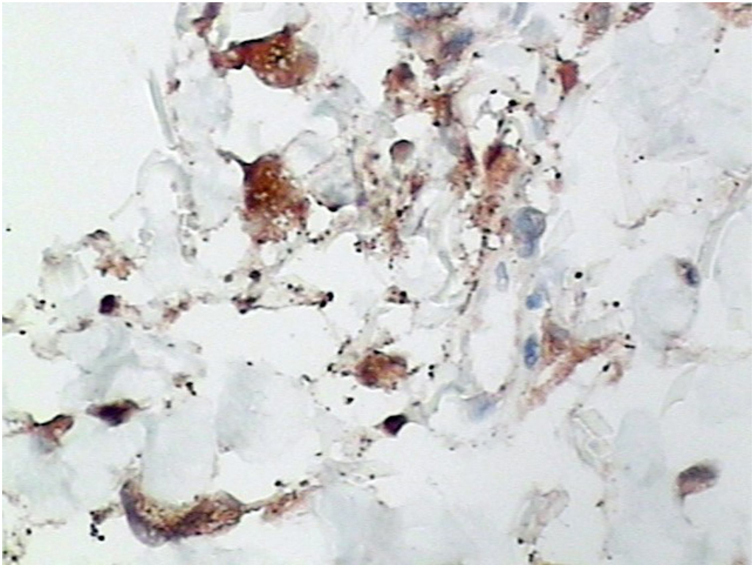
Immunohistochemistry study. Positive for CD68 (×400).

Punch biopsies of the skin lesions were performed, which showed a normal epidermis and fibrohistiocytic proliferation with small vessels in the reticular dermis, displaying lumen vessels filled with prominent endothelial cells, involved with a perivascular inflammatory response composed by multinucleated histiocytic cells (MCs) and a few plasma cells. In the papillary dermis, fibroblastic proliferation and thickened collagen fibers were found (Figure 2, Figure 3) and numerous bizarre MCs with scalloped margins were found in the adjacent dermis. The immunohistochemical (IHC) panel performed found the following: S-100 protein (negative), FXIIIa (positive) in MCs, CD68 (positive) in MCs, CD34 and CD31 (positive) in small vessels, and CD4 (positive) in dermal lymphocytes (Figure 4, Figure 5). The final diagnosis was established as compatible with multinucleate cell angiohistiocytoma. Due to the benign nature of the illness, the patient chose not to undergo treatment.

## Discussion

The designation MCAH was originally introduced in 1985 by Smith and Wilson-Jones,^1^ representing a rare fibrohistiocytic vascular proliferation of unknown etiology that is reported three times more frequently in women, and commonly affects middle-aged (> 40 years) or elderly individuals.^2^, ^3^ To date, there are about 140 cases of MCAH reported in the indexed literature (PubMed/MEDLINE), and its prevalence is believed to be underestimated due to poor clinical recognition of this pathology.

Lesions are asymptomatic and are mostly located on the face and in the acral regions, although they have been reported in other locations, such as the trunk and, more rarely, the mucous membranes;^2^  they are usually unilateral and present as papules ranging from 2-15mm in diameter. They may appear as reddish, pink, violaceous, or brown papules with a slightly raised dome-shaped surface, or may be flat and smooth.^2^, ^4^ Bilateral involvement and even generalized forms have been documented in a few cases.^2^ MCAH lesions develop over weeks to months, with no tendency to spontaneous regression.^5^

Regarding the differential clinical diagnosis, special attention should be given to Kaposi's sarcoma, especially when the MCAH is presented as grouped papules.^6^ Acroangiodermatitis, annular granuloma, angiofibroma, dermatofibroma, microvenular hemangioma, lichen planus, lymphocytoma, and reaction to insect bite should also be included.^7^

Although the etiology and pathogenesis of MCAH are still unknown, among the hypotheses, the predominant understanding is that lesions arise from a reactive rather than a neoplastic process. This hypothesis is supported by the fact that it is a benign entity with indolent behavior, the absence of extracutaneous involvement or malignant transformation, and the possibility of spontaneous regression is compatible with a reactive rather than neoplastic inflammatory process.^3^, ^8^, ^9^

Another theory proposes female hormonal influence on pathogenesis, noting the identification of estrogen receptor (ER) alpha expression in interstitial and multinucleated cells; this factor would explain the higher frequency in women. However, the identification of ER positivity has not been consistent in other reported cases.^3^, ^8^

In 2015, Frew published a review of 142 cases of MCAH. The author hypothesized that although MCAH has an initial inflammatory and vascular origin, histopathological events associated with fibrosis and atrophy play a key role in the pathogenesis of the disease, especially regarding the progression to multiple lesions.^10^

The main histopathological finding in MCAH is the proliferation of venules and capillaries in the dermis, accompanied by lymphocytic infiltrate and angular multinucleated cells. These cells may exhibit up to ten hyperchromatic nuclei and have basophilic cytoplasm; the cells express vimentin and factor XIIIa, and there is dermal fibrosis and sparse lymphohistiocytic infiltrate.^2^, ^7^, ^10^

The histological findings of MCAH are similar to those of several benign skin tumors and other fibroangiomatous conditions, which has led some authors to question the status of MCAH as an independent histopathological entity.^6^, ^10^ Some authors consider MCAH as a variant of dermatofibroma, with prominent vascular component and peculiar multinucleated cells.^10^ Other histological differential diagnoses include Kaposi's sarcoma, angiofibroma, lymphocytoma cutis, lichen planus and angiolymphoid hyperplasia with eosinophilia.

Differential diagnosis with Kaposi's sarcoma (KS) is important, because there is great clinical similarity between the entities, but there are some histopathological differences.^6^, ^8^, ^10^ Microscopically, KS consists of irregular anastomotic vascular channels and sieve-like chinks, extravasation of red blood cells, hemosiderin deposits, and spindle-shaped endothelial cells.^3^ KS cells express podoplanin, a marker of the lymphatic endothelium, which is not expressed by MCAH endothelial cells. Confirmation of human herpes virus 8 (HHV8) positivity (demonstrated with IHC or *in situ* hybridization) allows the distinction of KS from MCAH.^3^, ^10^

In most reported cases of MCAH, the IHC panel performed included staining for factor VIII, factor XIIIa, CD31, CD34, CD68, and vimentin in both the vascular endothelium and MCs. In Frew's review article, IHC showed 60% CD68-stained vascular endothelial cells.^10^ As expected, endothelial cells also expressed factor VIII staining, CD31, and CD34, and in the present case, CD31 and CD34 markers were also positive in these cells. Frew noted that MCs were negative in staining for endothelial markers, factor VIII, and CD34, with approximately half of the cases positively staining macrophages/histiocytes with factor XIIIa and CD68.^10^ Factor XIIIa and CD68 were also noted in the MCs of the present case.

MCAH treatment is generally not required because of the benign nature of the condition, except for esthetic reasons. Lesions can be treated with intralesional corticosteroids, surgical excision, cryotherapy, argon laser, intense pulsed light, and CO_2_ laser. Recently, a case treated with pulsed dye laser was reported, with good result.^3^, ^5^

The present report adds another case of MCAH to the literature, highlighting the difficulty of diagnosing this condition in dermatological practice. It is important to establish the correct diagnosis for this clinical condition and, especially, to exclude more serious diseases such as KS, mainly in the scenario of patients with AIDS or organ transplantation.

## Financial support

None declared.

## Authors’ contributions

Anderson Alves Costa: Drafting and editing of the manuscript.

Glaucia Ferreira Wedy: Conception and planning of the study.

Walter Belda Junior: Intellectual participation in the propaedeutic and/or therapeutic conduct of the studied cases.

Paulo Ricardo Criado: Drafting and editing of the manuscript; intellectual participation in the propaedeutic and/or therapeutic conduct of the studied cases; critical review of the literature; critical review of the manuscript.

## Conflicts of interest

None declared.
